# The effect of downstream translocation on Atlantic salmon *Salmo salar* smolt outmigration success

**DOI:** 10.1111/jfb.15928

**Published:** 2024-10-12

**Authors:** Ruaidhri Forrester, Hannele M. Honkanen, Jessie Lilly, Amy Green, Jessica R. Rodger, Brian A. Shields, Philip Ramsden, J. Peter Koene, Melanie Fletcher, Colin W. Bean, Colin E. Adams

**Affiliations:** ^1^ Scottish Centre for Ecology and the Natural Environment, School of Biodiversity, One Health and Veterinary Medicine University of Glasgow Glasgow UK; ^2^ Atlantic Salmon Trust Perth UK; ^3^ Environment Agency Penrith UK; ^4^ Natural England Penrith UK; ^5^ NatureScot Clydebank UK

**Keywords:** acoustic telemetry, Atlantic salmon, management, migration, smolts

## Abstract

Trap and transport, the capture and subsequent translocation of fish during the freshwater phase of their migration, is becoming more common as a management intervention. Although the technique can be successful, it is costly and can have unintended effects on the fish being transported. This study investigates whether trap and transport can be used to increase the migration success of Atlantic salmon, *Salmo salar*, smolts in naturally flowing rivers. Seaward‐migrating *S. salar* (*n* = 294) from two UK rivers were tracked using acoustic telemetric techniques. Outmigration success and timing were compared between non‐transported (released at the original in‐river capture site) and transported (released ca. 23 km downstream of the capture site) individuals. Downstream translocation increased the proportion of fish that successfully migrated to marine waters, and there was no indication that transport reduced post‐release survival. The post‐release migration speed of transported fish was slower than expected but this was likely a function of their advanced migration timing rather than an inhibition of their capacity to migrate. These results suggest that trap and transport can increase the outmigration success of *S. salar* smolts, but the earlier river exit dates of transported fish could negatively affect their survival at sea.

## 1 INTRODUCTION

The first seaward migration is risky for anadromous salmonids (Adams et al., 2022; Thorstad et al., 2012a). In the Atlantic salmon, *Salmo salar*, Linnæus 1758, there is evidence of considerable spatial and temporal variation in outmigration success (Rodger et al., 2024). For example, *S. salar* smolts, here defined as fish on their first seaward migration, have particularly high loss rates at river mouths (Kennedy et al., 2018; Vollset et al., 2016) and in estuaries (Dieperink et al., 2001; Jepsen et al., 2006). Low smolt migration success has also been observed in lentic habitats (Aarestrup et al., 1999; Honkanen et al., 2021), and this is almost certainly due to fish experiencing navigation difficulties in standing waters (Hanssen et al., 2021; Lilly et al., 2021). In modified river systems, dams can decrease migration success rates (Serrano et al., 2009), even where there are measures in place to assist passage (Havn et al., 2018). Indeed, the impoundment of water, rather than the barriers themselves, may drive some of these negative effects on migration success (Honkanen et al., 2021).

In an effort to reduce the impact of in‐river barriers or impoundments on downstream passage, some managers have attempted to increase migration success by transporting salmonid smolts around areas of suspected high loss. This process, frequently called trap and transport, involves capturing migrating fish at an upstream location, transporting the fish around the migration impediment, then releasing them closer to the sea. There are a number of well‐established trap‐and‐transport schemes, mainly for Pacific salmonids (*Oncorhynchus* spp.) (Kock et al., 2021), but also for *S. salar* (Mills, 1994; Newton et al., 2021). Similar programs also exist for both the upstream and downstream translocation of other salmonid life‐history stages and species (Lusardi & Moyle, 2017).

The aim of trap and transport, when used as a management tool for salmonids on their first seaward migration, is to increase their migration success rate by circumventing high‐risk parts of the migration pathway. However, such intervention is not without potential costs. Barge‐transported chinook salmon, *Oncorhynchus tshawytscha* (Walbaum 1792), smolts have been shown to have higher pathogen infection rates (van Gaest et al., 2011) and lower survival post infection (Arkoosh et al., 2006) than in‐river migrants. Capture and confinement during the execution of the process can cause abrasions, and scale and mucus damage might, for example, inhibit osmoregulation (Zydlewski et al., 2010). Trap and transport could also increase predation risk by temporarily compromising the sensory capabilities of transported fish (Halvorsen et al., 2009) or by advancing the migration timing of a proportion of the migration group (Rechisky et al., 2012) and thereby decreasing co‐migrant density (Furey et al., 2016; Hvidsten & Johnsen, 1993). Sequential cues encountered during the seaward migration help adult *S. salar* to locate their spawning grounds on the return migration (Haraldstad et al., 2022); trapping and transporting smolts may thus decrease spawning success by disrupting this imprinting. Although the trapping of smolts during the smolt run may, in itself, have little impact on their subsequent migration (Sortland et al., 2024), transport can increase stress in smolts (Nomura et al., 2009) and strong primary stress responses are correlated with lower at‐sea survival (Virtanen et al., 1991). However, transport‐induced stress can be mitigated by anesthetizing fish or by transporting them in brackish water (Finstad et al., 2003).

For a trap‐and‐transport scheme to be successful as a management intervention, transported fish need to have a higher adult return rate than those that migrated to sea naturally. However, given the potential impacts of the intervention, this aim may not be achieved in all cases. Hence, the magnitude of any positive effect of trap and transport, and the circumstances in which management net gains are achieved, are active research fields. To date, the most studied trap‐and‐transport system is in the Columbia River basin in North America. There, *O. tshawytscha* and steelhead, *Oncorhynchus mykiss* (Walbaum 1792), smolts are regularly transported up to 470 river km (over ca. 3 days) to bypass up to eight dams (Rechisky et al., 2012; Ward et al., 1997). Between 1968 and 1989, when fish were transported in either trucks or barges, an initial assessment showed that the scheme did not consistently increase adult return rate (Ward et al., 1997). However, a more recent synthesis (1994 to 2020), based entirely on the use of barges, found that transport did increase adult return rate (McCann et al., 2022). The elevated return rate of transported fish in this system appears to be primarily driven by the high rate of transport success: 98% of fish survived transport in barges (Budy et al., 2002; McMichael et al., 2011) while only 48% successfully migrated in the river from the capture site to the barged fish release site (Widener et al., 2023). Despite the positive effect of trap and transport on overall adult returns, migration success can be lower for transported than non‐transported fish in sections of both the juvenile seaward (Eder et al., 2009) and adult spawning (McCann et al., 2022; Schaller et al., 2014) migrations. Hence, at times, there appear to be delayed effects of trap and transport that decrease the migration success of transported fish.

In contrast to Pacific salmonid rivers, there has been little research on the effectiveness of trap and transport in relatively small, naturally flowing *S. salar* watersheds. Mills (1994) recorded a higher adult return rate for transported than non‐transported *S. salar* smolts in the River Bran, Scotland, while Axford (1991) recaptured too few individuals to compare survival between transport treatments in the River Ouse, England. Babin (2019) found that downstream translocation did not affect the subsequent, post‐release migration success of *S. salar* smolts in the Saint John River, Canada, but this trap‐and‐transport scheme was unusual in that fish were transported months before they started to migrate naturally. In the present study, acoustic telemetric techniques were used to determine the differential migration success of trapped and transported and naturally migrating *S. salar*. The study was replicated across two UK rivers, and in each study area, transported fish bypassed a natural lake. We used this approach to evaluate support for five specific null hypotheses:

1. Transport does not affect migration success from the capture site to the transported fish release site.
2. Transport does not affect migration success from the transported fish release site to the outer limit of the estuary.
3. Transport does not affect migration success from the capture site to the outer limit of the estuary.
4. There is no difference between the dates that non‐transported and transported fish exit the river and the nearshore marine zone.
5. There is no difference between the migration speeds of non‐transported and transported individuals downstream of the transported fish release site.

## 2 METHODS

### 2.1 Study areas and field methods

**FIGURE 1:**
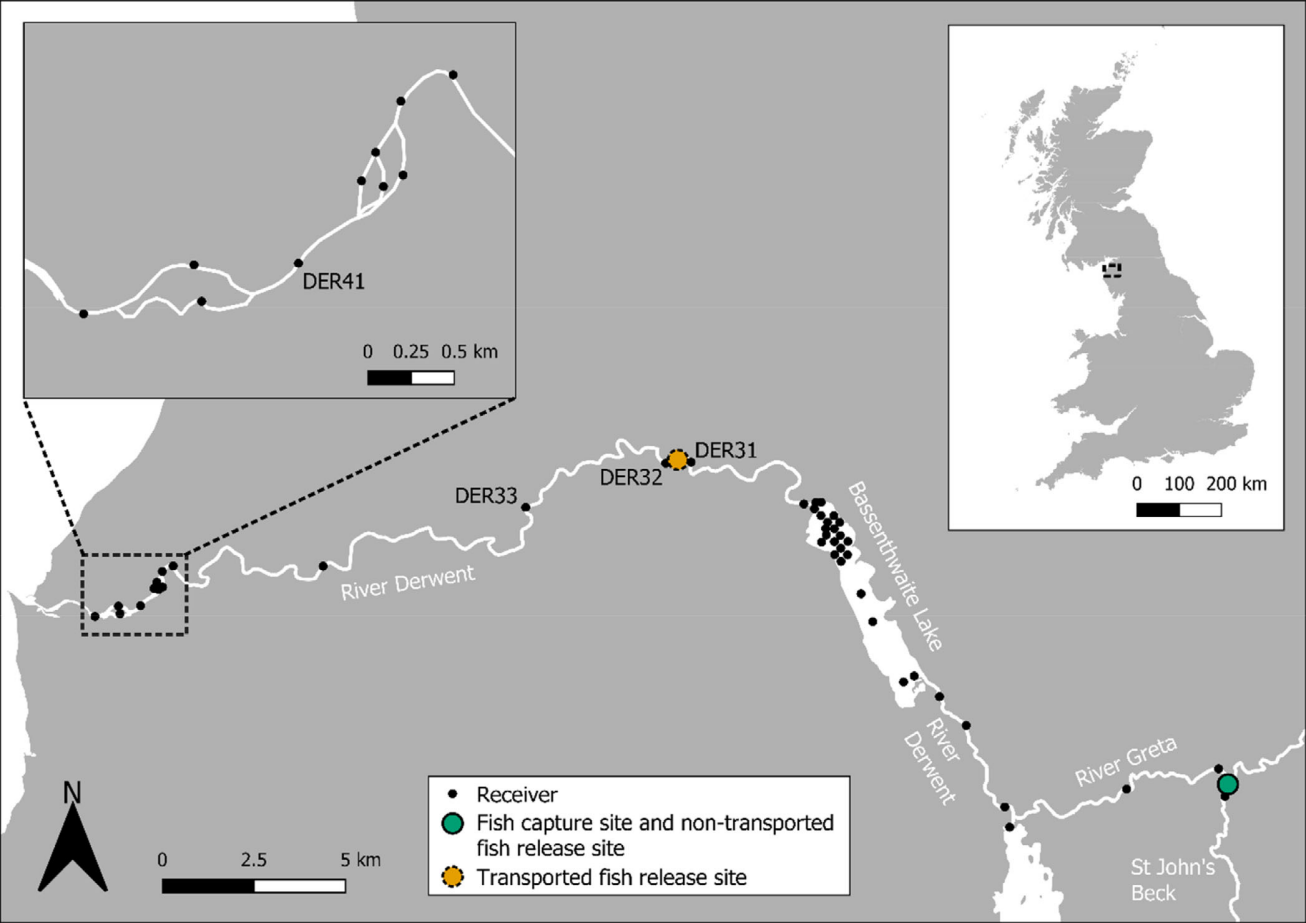
The River Derwent study area. Selected receiver names are in black text and waterbody names are in white text. All tagged *Salmo salar* smolts were captured by a rotary screw trap deployed at the fish capture site (green dot). This figure contains public sector information licensed under the Open Government License v3.0. Contains OS data © Crown copyright and database right 2021.

**FIGURE 2:**
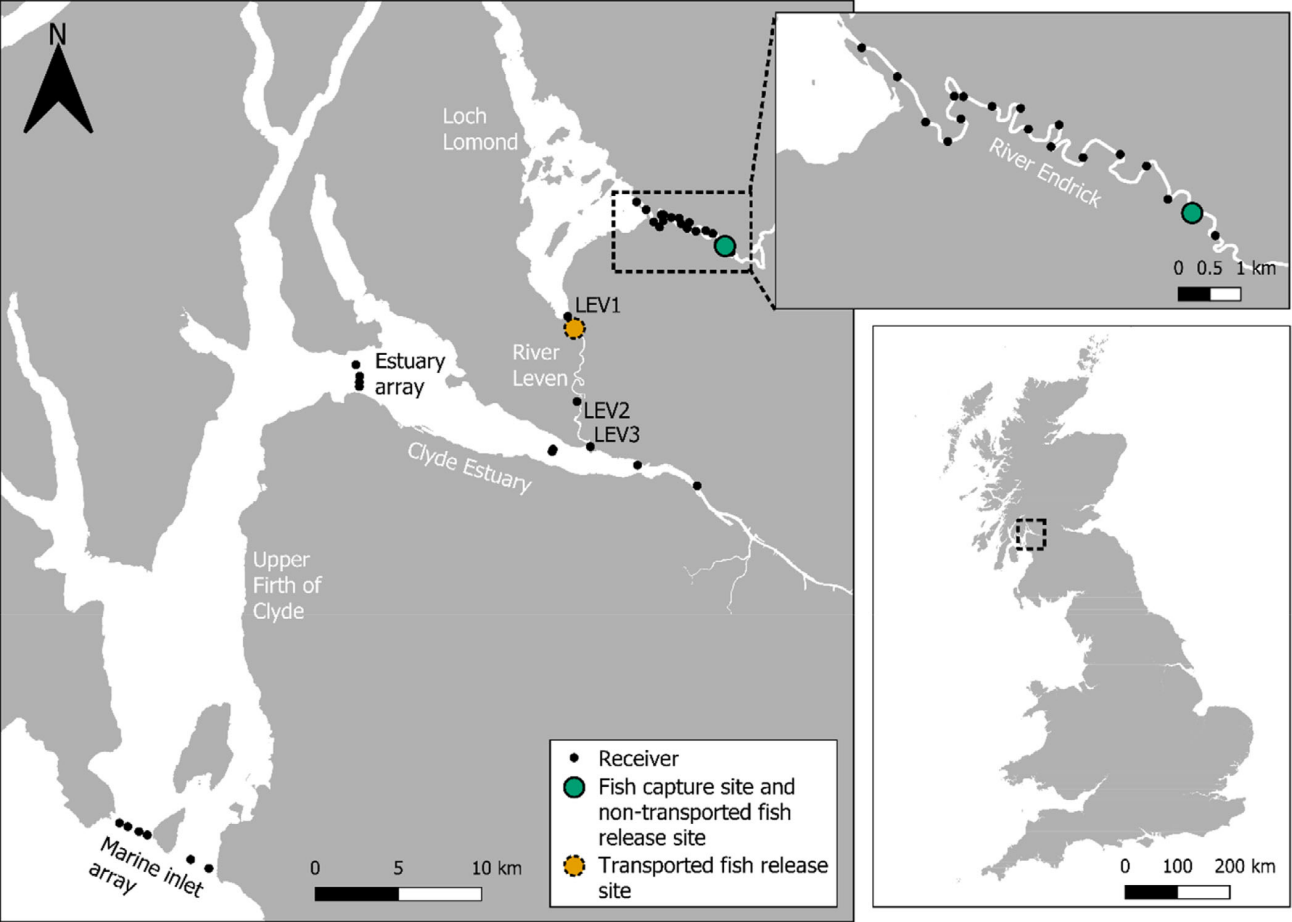
The Lomond study area. Selected receiver names are in black text and water body names are in white text. All tagged *Salmo salar* smolts were captured by a rotary screw trap deployed at the fish capture site (green dot). Only receivers recovered after deployment are plotted. Contains public sector information licensed under the Open Government License v3.0. Contains OS data © Crown copyright and database right 2021.

*Salmo salar* smolts were tracked in two study areas: the River Derwent system, north‐west England, and the Lomond system, central‐west Scotland. The Derwent study area covered 48.4 km of freshwater (Figure 1) including rivers and Bassenthwaite Lake (5.2 km^2^ surface area, 5.3 m mean depth, 19 m maximum depth; UK Centre for Ecology and Hydrology, 2023). The Lomond study area consisted of a minimum 34 km freshwater migration distance, comprising 12.6 km of the lower Endrick Water, Loch Lomond (71.2 km^2^ surface area, 37.0 m mean depth, 189.9 m maximum depth; Murray & Pullar, 1910), and the River Leven (12 km length) as well as a section of the Clyde Estuary and Firth of Clyde (ca. 47 km in length from the mouth of the River Leven; Figure 2). The River Derwent and the Endrick Water are both designated as Special Areas of Conservation (SAC) under the Habitats Directive 1992, with *S. salar* being a feature of interest at both sites.

#### 2.1.1 Smolt tracking and transport

*S. salar* smolts were tracked using acoustic telemetric techniques, an established method of collecting fish movement data (Hussey et al., 2015). The approach involves implanting acoustic transmitters within the body cavities of migrating individuals, then deploying hydrophone receivers across a study area to detect tagged fish as they pass. Movements can be inferred from patterns of transmitter detections across receivers.

A 1.2‐m diameter rotary screw trap was deployed at each of the study areas (in the Derwent system at 54°36′38.1″N, 3°3′42.2″W and in the Lomond system at 56°02′57.1″N, 4°26′23.8″W) for the whole of the salmon smolt migration period (approximately March to May). To reduce the effects of tag burden and tag expulsion on survival (Brunsdon et al., 2019), only fish ≥130 mm fork length and ≥20 g wet mass were tagged with acoustic transmitters. In the Derwent study area, 150 *S. salar* smolts (mean fork length ± SD = 141.62 ± 8.09 mm, mean wet mass ± SD = 29.58 ± 5.37 g) were tagged. In the Lomond study area, 144 *S. salar* smolts (mean fork length ± SD = 143.05 ± 9.72 mm, mean wet mass ± SD = 29.77 ± 6.09 g) were tagged. Two transmitter tag types were used: Innovasea V7‐2x and V7‐2L (Innovasea). In the Derwent system, 35 V7‐2x (50‐day expected lifespan) and 115 V7‐2L (75‐day expected lifespan) transmitters were deployed, while in the Lomond system 144 V7‐2x transmitters were used, of which 97 had a 64‐day expected lifespan and 47 had a 75‐day expected lifespan.

Acoustic transmitters were surgically implanted into fish. Fish were anesthetized with a buffered (to ca. pH 7) river water solution of 0.1 g L^−1^ tricaine methanesulphonate (MS222) and 0.1 g L^−1^ sodium bicarbonate. Each fish was sedated to level 3 anesthesia (loss of upright position, reduced gill movement, and unresponsiveness to touch) before surgery. An incision was made next to the ventral keel, approximately 3–4 mm anterior to the pelvic girdle, and the transmitter was inserted into the peritoneal cavity. Each incision was closed with a pair of 4/0 Ethilon nylon sutures (Ethicon Ltd.). During surgery, the gills were constantly irrigated, alternating between river water and a 50% concentration of MS222 solution depending on the level of anesthesia. After tagging, all fish were placed in an aerated recovery container for 20 min. All fish were then inspected and assessed for recovery from the tagging process by an experienced researcher.

Each tagged fish was randomly assigned to either a non‐transported or transported migration group. Of the 150 fish tagged in the Derwent, 92 were released immediately below the rotary screw trap (non‐transported) and 58 were transported approximately 25 km (river distance) downstream to a release site at 54°41′15.6″N, 3°17′52.2″W (Figure 1). In the Lomond system, 98 tagged fish were released just below the trap in the Endrick Water (non‐transported), while 46 were transported and released in the River Leven at 56°0′30.8″N, 4°35′23.2″W approximately 21 km (river distance) downstream (Figure 2). During transport by van, fish in the Lomond study area were held in 60 liter bags, 30% filled with river water. Oxygen gas was added to each bag before it was sealed and the bags were placed in large plastic buckets for extra stability. Fish density did not exceed ca. 0.5 fish per liter. Derwent study area fish were transported in boxes filled with river water and aerated with aquarium pumps. In both study areas transport lasted ca. 20 min and transported fish bypassed a large lake. Before release, both non‐transported and transported fish were given a minimum of 30 min of in‐river recovery in a submerged cylindrical nylon cage (45 cm diameter, 45 cm height). Non‐transported smolts were released consecutively in groups while those transported were released once per day. After accounting for transport time and the post‐transport recovery period, transported fish were released approximately 60 min after the last non‐transported fish on a given tagging day. The dates on which fish were tagged and released are summarized in Table 1 and listed fully in Appendix S1. Non‐transported and transported fish had statistically similar fork lengths (Welch's *t*‐test, *t* = −0.304, *df* = 228.14, *p* = 0.762).

**TABLE 1:** The release dates of *Salmo salar* smolts tagged with acoustic transmitters.

Study area	Migration route	Median release date (range)	Number of release dates
Derwent	Non‐transported	May 3, 2021 (April 14 to May 5)	10
Derwent	Transported	April 27, 2021 (April 20 to May 4)	6
Lomond	Non‐transported	April 27, 2021 (April 15 to May 4)	15
Lomond	Transported	April 27, 2021 (April 23 to April 30)	7

#### 2.1.2 Ethical statement

Rotary screw trapping was licensed by the relevant authorities (Derwent: the Environment Agency; Lomond: Marine Scotland). In compliance with UK animal welfare legislation, all tagging was conducted under UK Home Office license PPL 70/8794.

#### 2.1.3 Acoustic receivers

To detect transmitters and thereby track tagged fish, acoustic receivers were deployed across each study area. In the Derwent system, 44 acoustic receivers (17 VR2W and 27 VR2TX; Innovasea; Figure 1) were in place between March 25 and July 15, 2021. In the Lomond system, receivers were deployed at each of 37 locations in freshwater and the marine estuary (6 VR2W, 21 VR2TX, 10 VR2AR; Innovasea; Figure 2) from March 16 to July 12, 2021. Three Lomond system receivers were lost following deployment (one in the estuary array, two in the marine inlet array); these are not shown on Figure 2.

### 2.2 Data cleaning

As false detections are common in acoustic telemetry systems (Pincock, 2012), a standardized cleaning process was applied to all detection data to improve their reliability. First, detections with an identification code that did not match that of any deployed transmitter were removed. Then, detections were removed if they were a repeat detection of a transmitter within a period less than the minimum inter‐transmission delay (V7‐2x < 18 s, V7‐2L < 17.5 s). Finally, to ensure that the movements of all tracked fish were plausible, the locations of individual fish detections were plotted against time and the resulting graphs were visually inspected.

### 2.3 Migration success

#### 2.3.1 Capture site to transported fish release site

When measuring migration success between the capture site and the transported fish release site, transported fish were determined as “successful” if they were behaving normally immediately before they were released. Non‐transported fish were “successful” if they were detected by a receiver at, or downstream of, the transported fish release site (for Derwent detections on or beyond DER31 and DER32 [Figure 1], for Lomond detections on or beyond LEV1 [Figure 2]). Fish that did not meet either of these conditions were regarded as “unsuccessful”. The success rate was expressed as the number of successful fish as a proportion of all tagged fish of a given migration route (the migration routes were transported and non‐transported).

#### 2.3.2 Migration success downstream of the transported fish release site

To investigate whether there was differential migration success downstream of the transported fish release site, transported and non‐transported fish success rates were compared in three sections of the study areas. Each started at the transported fish release site but differed in its endpoint. The first section ended at the next receiver downstream (i.e. DER33 in the Derwent [Figure 1], LEV2 in the Lomond system [Figure 2]). The second ended at the final freshwater receiver with 100% detection efficiency (DER41 in the Derwent [Figure 1], LEV3 in the Lomond system [Figure 2]; see Appendix S2 for detection efficiencies). The third, which applied to the Lomond study area only, ended at the outer limit of the Clyde Estuary (estuary array [Figure 2]). For each section, fish detected at or beyond the endpoint of that section were deemed successful migrants. Unsuccessful individuals were those that were recorded at the start of that section but not detected at or beyond the section end.

#### 2.3.3 Overall migration success

Overall migration success, measured from the capture site onwards, was calculated for transported and non‐transported fish. Fish were determined to have successfully migrated to the river mouth if detected at DER41 in the Derwent system (Figure 1) or LEV3 (or downstream receivers) in the Lomond system (Figure 2), otherwise they were deemed unsuccessful. At the Lomond study area, migration success to the outer limit of the Clyde Estuary was determined as a fish that was detected at or beyond the estuary array (Figure 2).

#### 2.3.4 Migration success comparison statistics

For each migration success measurement taken at both study areas, a 2 × 2 × 2 frequency table was constructed to tabulate the number of successful or unsuccessful fish for each migration route and study area. A Breslow–Day test of homogeneity with Tarone's adjustment tested whether the effect of transport on success depended on study area. If the Breslow–Day test indicated no interaction, then a Cochran–Mantel–Haenszel test evaluated support for the null hypothesis that equal proportions of transported and non‐transported fish were successful. Otherwise, the proportion of fish that migrated successfully was compared between transported and non‐transported categories for each study area separately with a chi‐squared test of homogeneity with Yates' continuity correction. For the Lomond‐only migration success measurements, a chi‐squared test of homogeneity with Yates' continuity correction tested whether the proportion of successful migrants varied between transported and non‐transported migration routes. All expected frequencies were >3.

### 2.4 Migration timing

#### 2.4.1 River exit timing

An initial linear model that included all explanatory variables that might putatively affect river exit timing was fitted by maximum likelihood. The response variable, river exit date, was the day of the year (DOY) that a fish was first detected by the final freshwater receiver with 100% detection efficiency (either DER41 or LEV3; see Appendix S2 for detection efficiencies). The explanatory terms included in this initial model were migration route (transported or non‐transported), study area (Derwent or Lomond), and release DOY (continuous) and all possible pairwise interactions between these variables. Next, stepwise backward model selection was used to identify the most parsimonious river exit timing model. Starting with the initial model, a nested model was specified by dropping one explanatory term. A likelihood ratio test (LRT) was then used to assess the probability that the more complex model and the simpler nested model fitted the data equally well. If *p* > 0.05, then the simpler nested model was adopted as the provisional best fit and the dropped variable was discarded. Otherwise, the more complex model was the provisional best fit and the dropped variable was retained. This process was iterated until all explanatory variables had been either discarded or retained. The retained explanatory variables comprised the final model and the explanatory contribution of each of these was inferred by an LRT between the final model and a nested model that excluded the variable in question. Diagnostic plots of each model's residuals were inspected to ensure that model assumptions were met.

#### 2.4.2 Marine inlet exit timing

At the Lomond study area, marine inlet exit times were determined as the first detection of an individual by the marine inlet receiver array (Figure 2). Other than the omission of study area and its interactions with other variables, marine inlet exit timing was analyzed with the same modeling framework as river exit timing.

### 2.5 Migration speed

Migration speed (km day^−1^) was calculated for each fish by dividing the river distance from the transported fish release site to the final freshwater receiver by the time taken to move between these two points. Migration speed was then analyzed with two linear models fitted by maximum likelihood. The response variable of both models was the natural logarithm of migration speed. The explanatory variables of the first model were migration route, study area, and an interaction between these two terms. In addition to migration route and study area, the second model also included a main effect of the date (DOY) of arrival at the transported fish release site and all possible pairwise interactions between these three variables. For each of these two initial complex models, the best fitting explanatory variables were identified by the backwards selection process described above.

### 2.6 Software

All data analyses were conducted in R (R Core Team, 2021). Breslow–Day tests were performed using the “DescTools” package (Signorell et al., 2022). Bar plots and boxplots were compiled with “ggplot2” (Wickham, 2016). Linear model main effects were plotted with “jtools” (Long, 2022) and interactive effects were plotted with “interactions” (Long, 2019). Maps were produced in QGIS (QGIS Development Team, 2023).

## 3 RESULTS

### 3.1 Migration success

**FIGURE 3:**
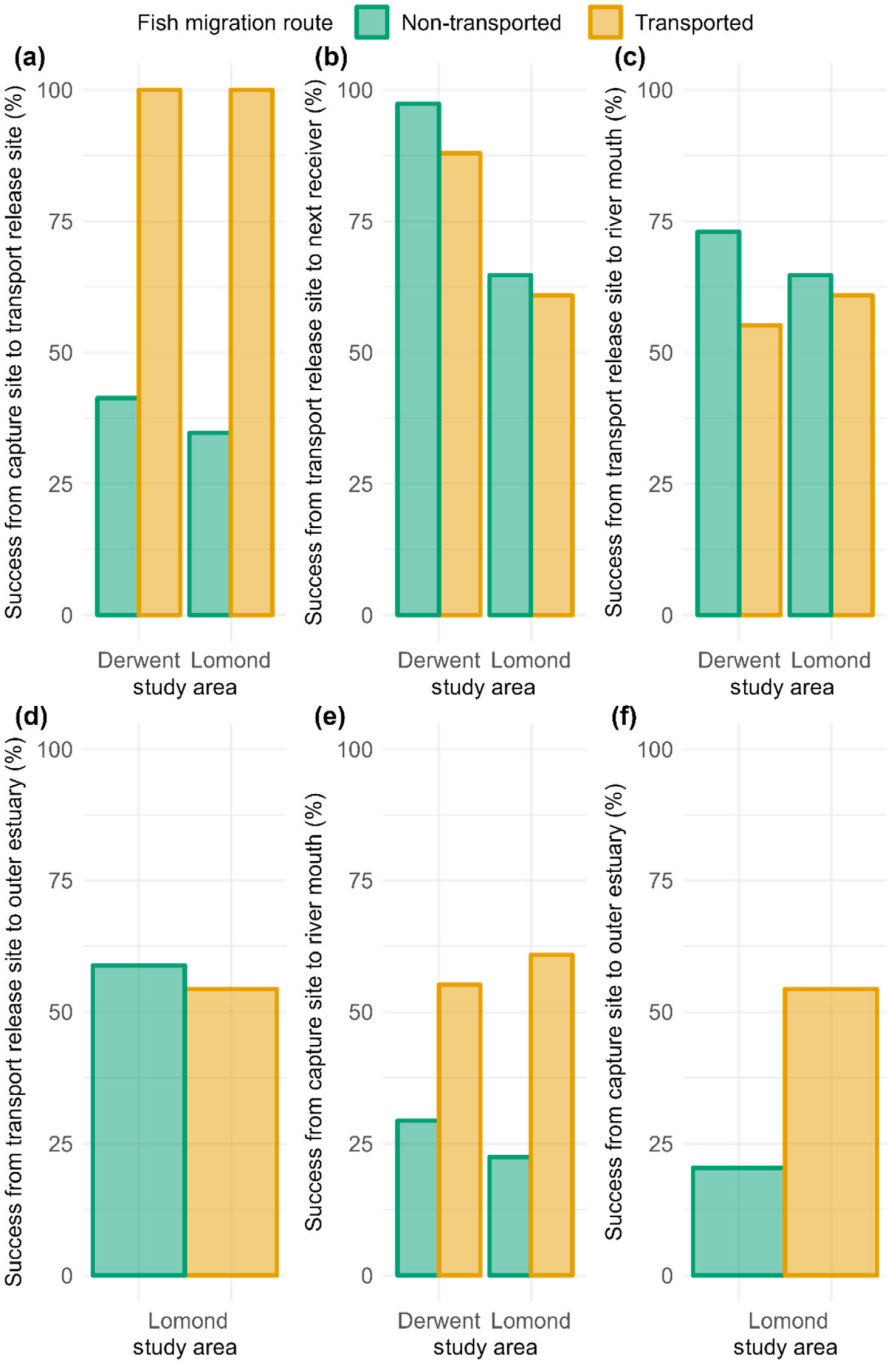
*Salmo salar* smolt migration success (a) from the capture site to the transported fish release site, (b) from the transported fish release site to the next receiver downstream, (c) from the transported fish release site to the river mouth, (d) from the transported fish release site to the outer estuary (Lomond only), (e) from the capture site to the river mouth, and (f) from the capture site to the outer estuary (Lomond only). For a given migration section and fish migration route, migration success was calculated as the number of successful migrations expressed as a proportion of the number of attempted migrations.

Non‐transported and transported smolts had different migration success rates between the capture site and the transported fish release site. While all fish survived transport, non‐transported smolt migration success was relatively low in this section (Table 2 and Figure 3a).

**TABLE 2:** The migration success rates of transported and non‐transported *Salmo salar* smolts.

Migration section	Derwent migration success (%)		Lomond migration success (%)		*p* value
	Non‐transported	Transported	Non‐transported	Transported	
Capture site to transport release site	41.3	100	34.7	100	‐
Transport release site to next receiver downstream	97.4	87.9	64.7	60.9	0.340
Transport release site to river mouth	71.1	55.2	64.7	60.9	0.221
Transport release site to outer estuary^a^	‐	‐	58.8	54.3	0.864
Capture site to river mouth	29.3	55.2	22.4	60.9	<0.001
Capture site to outer estuary^a^	‐	‐	20.4	54.3	<0.001

*Note*: The *p* values are from Cochran–Mantel–Haenszel tests of the null hypothesis that across both study areas equal proportions of transported and non‐transported fish were successful. For the Lomond‐only migration success measurements, the *p* values are from a chi‐squared test of homogeneity. Migration success rates were determined as the number of individuals that reached the end of a migration section as a proportion of the number that started the section.

^a^ Denotes migration sections that were only observed at the Lomond study area.

Downstream of the transported fish release site, non‐transported and transported individuals had similar migration success rates to the next receiver downstream (*χ* ^2^ = 0.911, *df* = 1, *p* = 0.340; Table 2 and Figure 3b) and to the river mouth (*χ* ^2^ = 1.498, *df* = 1, *p* = 0.221; Table 2 and Figure 3c). These effects were consistent across both study areas (next receiver downstream, *χ* ^2^ = 1.668, *df* = 1, *p* = 0.197; river mouth, *χ* ^2^ = 0.664, *df* = 1, *p* = 0.415). In the Lomond system, where offshore movements could be observed, non‐transported and transported fish also had similar migration success rates from the transport release site to the outer estuary (*χ* ^2^ = 0.029, *df* = 1, *p* = 0.864; Table 2 and Figure 3d).

Overall migration success, measured from the capture site to the river mouth, was markedly higher for transported than for non‐transported fish (Derwent 88.3% higher, Lomond 171.9% higher; *χ* ^2^ = 27.346, *df* = 1, *p* < 0.001; Table 2 and Figure 3e). This effect did not vary between study areas (*χ* ^2^ = 1.305, *df* = 1, *p* = 0.253). The proportion of Lomond fish that successfully made the full migration from the capture site to at least the outer limit of the Clyde estuary was also different between migration routes (*χ* ^2^ = 15.242, *df* = 1, *p* < 0.001), with transported individuals having the higher success rate (Table 2 and Figure 3f).

### 3.2 Migration timing

#### 3.2.1 River exit timing

**FIGURE 4:**
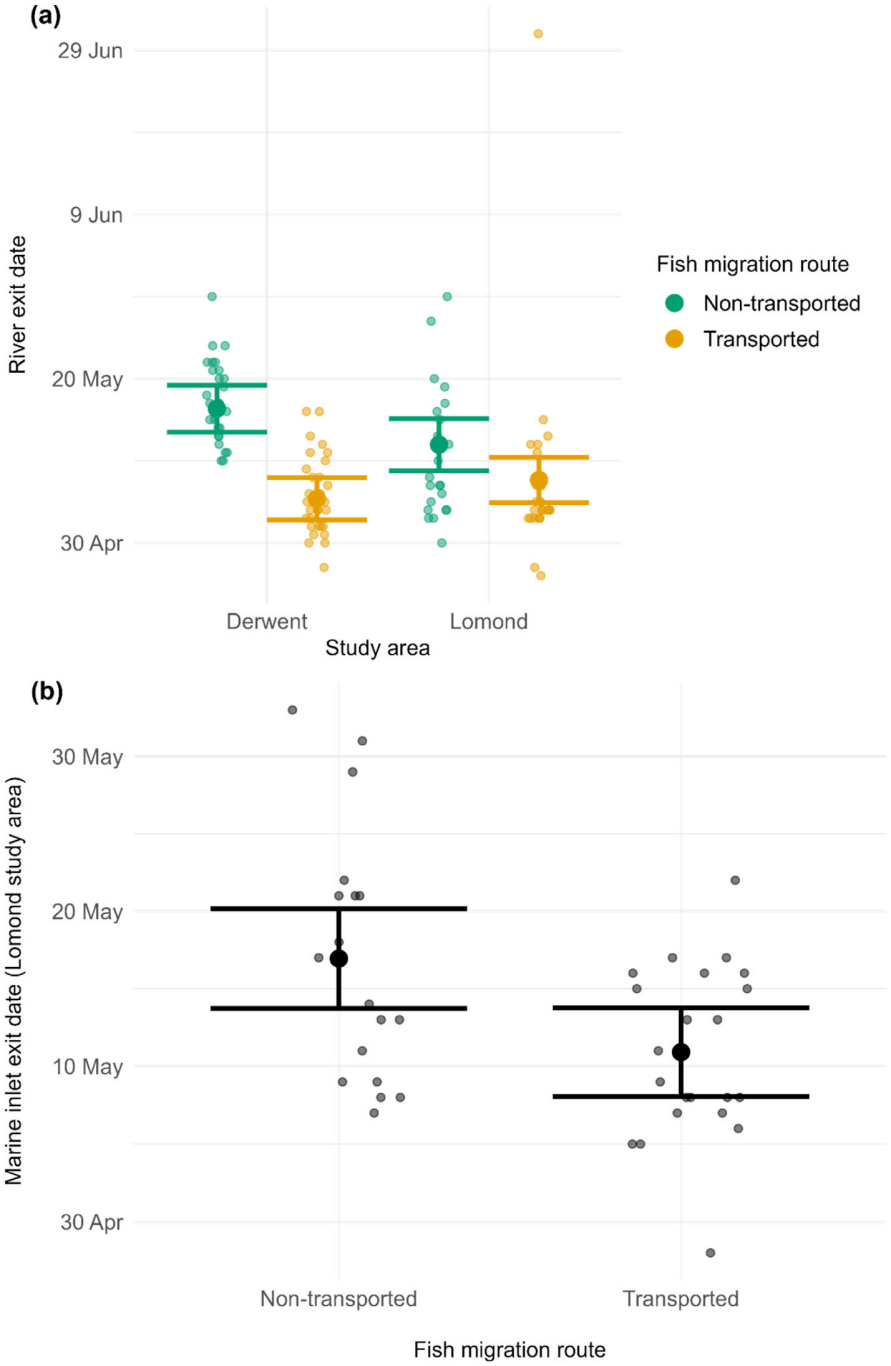
*Salmo salar* (a) river and (b) marine inlet exit dates. All fish were on their first seaward migration. The small points are observed exit dates. Random horizontal variation has been added to these points to minimize overplotting. The large points are model‐predicted exit dates and the lines are 95% confidence intervals. Predicted river exit dates are from a linear model with release date and a site‐migration route interaction as fixed effects. Predicted marine inlet exit dates are from a linear model with migration route as a fixed effect. All marine inlet exit timing data are from the Lomond study area. All dates are 2021.

In the Derwent and Lomond study areas respectively, 59 (27 non‐transported, 32 transported) and 50 (22 non‐transported, 28 transported) fish were detected by the final freshwater receivers. The final model for river exit date included an interaction between migration route and study area (*χ* ^2^ = 5.526, *df* = 1, *p* = 0.019), and a main effect of release DOY (*χ* ^2^ = 12.132, *df* = 1, *p* < 0.001). In both study areas, transported fish exited the river earlier than those not transported, but the magnitude of the effect varied between systems. Derwent and Lomond transported fish were predicted to exit the river 10.98 and 4.30 days, respectively, earlier than those not transported (Figure 4a). For the fish released on the mean date across both study areas (April 27), the model‐predicted river exit dates in the Derwent system were May 5 for transported fish and May 16 for non‐transported fish, while in the Lomond system they were May 7 and May 11 for transported and non‐transported fish, respectively. The effect of fish release date on river exit date was a positive one, thus the river exit date was predicted to increase by 0.50 days for each day later a fish was released.

#### 3.2.2 Marine inlet exit timing

In the Lomond system, 41 (18 non‐transported, 23 transported) fish were detected by the marine inlet receiver array (Figure 2). Migration route was the only explanatory variable in the final model (*χ* ^2^ = 7.681, *df* = 1, *p* = 0.005) and transported fish were predicted to exit the inlet 6.03 days earlier than non‐transported fish. Model‐predicted inlet exit dates were May 10, 2021 and May 16, 2021 for transported and non‐transported fish, respectively (Figure 4b).

### 3.3 Migration speed

**FIGURE 5:**
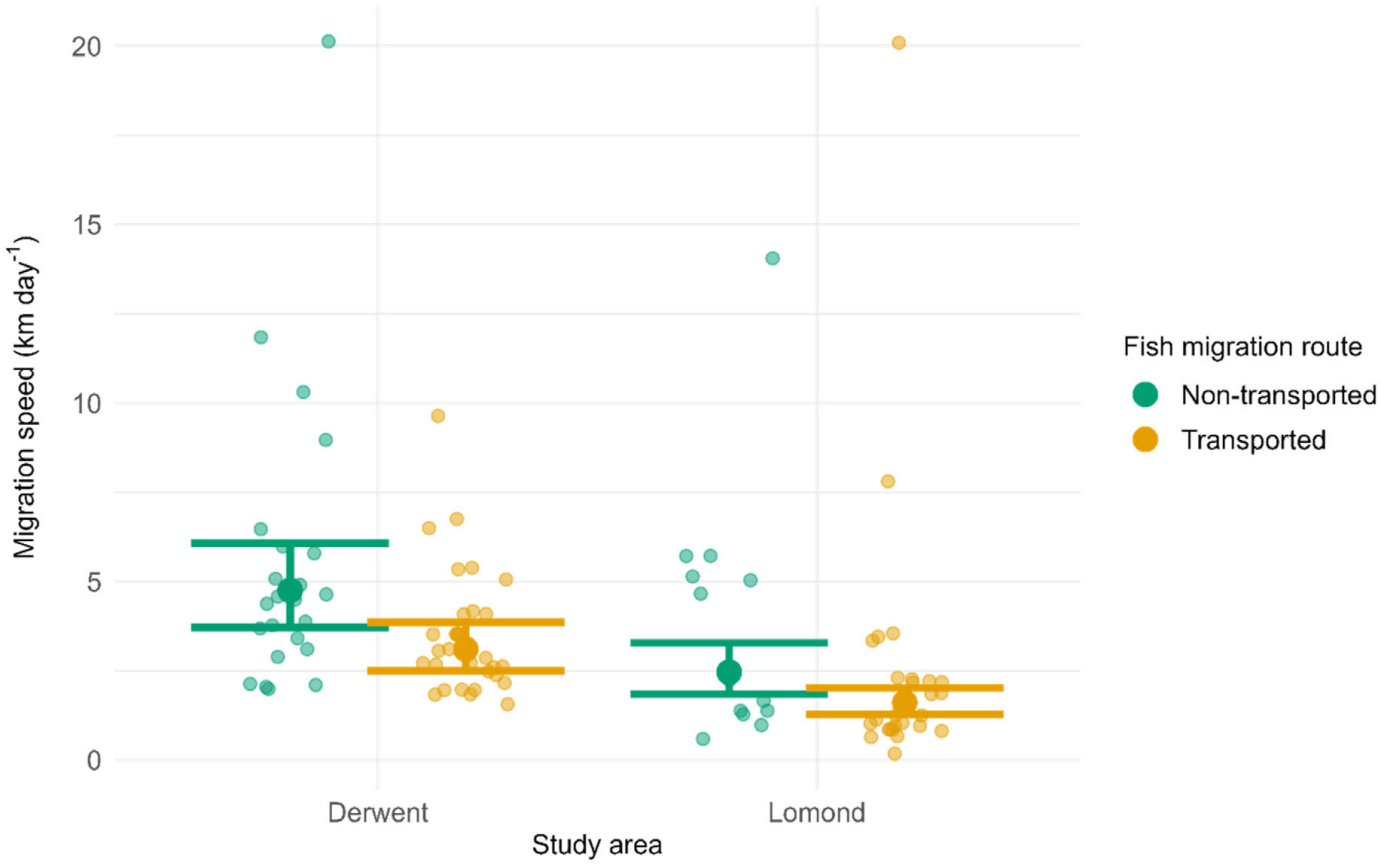
*Salmo salar* smolt migration speeds between the transported fish release site and the river mouth. The small points, whose locations have been randomised horizontally, mark observed fish migration speeds. The large points are predictions from a linear model with the natural logarithm of migration speed as the response variable and migration route and study area as explanatory variables. The lines are 95% confidence intervals. Predicted migration speeds and confidence intervals were calculated on the natural logarithm scale then back transformed.

When the initial migration speed model did not include a measure of migration timing, the final model consisted of the main effects of migration route (*χ* ^2^ = 8.475, *df* = 1, *p* = 0.004) and study area (*χ* ^2^ = 19.867, *df* = 1, *p* < 0.001). This model predicted that migration speed from the transported fish release site to the river mouth (*n* = 96) would be slower for transported than non‐transported fish by a factor of 0.65 and slower for Lomond than Derwent fish by a factor of 0.52 (Figure 5). However, when the initial complex model included a measure of migration timing, namely the date of arrival at the transported fish release site, the final model included an effect of study area (*χ* ^2^ = 17.051, *df* = 1, *p* < 0.001) and a positive effect of migration timing (*χ* ^2^ = 13.306, *df* = 1, *p* < 0.001) but no effect of migration route. Therefore, transport did not have a marginal effect on migration speed when its advancive effect on migration timing was accounted for.

## 4 DISCUSSION

An ideal trap‐and‐transport scheme would have high in‐transport survival, either increase or have no effect on post‐release migration success, increase overall migration success, and have no effect on migration timing or migration speed. In this study, compared with non‐transported *S. salar* smolts on their first seaward migration, transported fish had higher migration success between the capture site and the transported fish release site, similar migration success downstream of the transported fish release site, and higher overall migration success. However, transported fish had earlier river and marine inlet exit times, and slower migration speeds downstream of their release site. Therefore, the trap‐and‐transport scheme increased outmigration success without inflating the post‐release migration failure rate of transported fish, but the intervention had the unintended effects of advancing river and marine inlet exit timing and slowing the speed of post‐release migration.

Across the two study areas, all fish survived transport. This is consistent with observations of 98% in‐barge survival of juvenile *O. tshawytscha* (Budy et al., 2002; McMichael et al., 2011) and 99.7% *S. salar* smolt survival during transport by road (Mills, 1994). The migration success of non‐transported fish from the capture site to the transported fish release site was relatively low (Derwent = 41.3%, Lomond = 33.7%). This was unsurprising because all non‐transported fish migrated through lakes, and standing waters are associated with erratic navigation (Lilly et al., 2021) and high vulnerability to predation (Jepsen et al., 2000). As migration success downstream of the transported fish release site was similar regardless of whether fish were transported or not, the strong positive effect of transport on overall migration success was largely driven by differential success between the capture site and the transported fish release site.

Transport increased overall migration success despite there being room for improvement in the design of the trap‐and‐transport scheme deployed here. Transport may have had a stronger positive effect on overall migration success if the transported fish were released at night (Glover & Stephen, 2023; Vollset et al., 2017), into the sea (Gunnerød et al., 1988) or estuary (Solazzi et al., 1991), and at the same time as non‐transported migrants were expected to arrive (Rechisky et al., 2012). Both the availability of appropriate holding facilities and the capacity to accurately predict the migration phenology of naturally migrating smolts would be needed to implement a synchronized release strategy. The increased cost and logistical complexity of these measures could be minimized by only transporting fish in years when environmental conditions are expected to have a strong negative effect on freshwater survival. Therefore, the results presented here are encouraging, and there are feasible modifications that could further improve the effectiveness of the technique as a possible management intervention.

Transport advanced migration timing in both the Derwent and Lomond systems but the magnitude of this effect varied between the two study areas. Transport decreased river exit date by 11.0 days in the Derwent system compared with a decrease of 4.3 days in the Lomond system. This differential effect size was surprising given that in both systems non‐transported fish took a similar number of days to move from the capture site to the transported fish release site (Derwent median [range] = 10.99 days [0.35–26.48], Lomond median [range] = 10.32 days [5.87–28.69]). The interaction might have been explained by differences in receiver layout between the Derwent and Lomond systems, but this hypothesis was not supported (for details, see Appendix S3). Given the high estuarine migration success rate of transported Lomond fish (Figure 3d), transported smolts appeared to be prepared for high salinity water despite their early migration. However, this might not necessarily be the case in trap‐and‐transport schemes where fish are transported over longer distances.

Between the transport release site and the river mouth, transported fish were predicted to have a slower migration speed than non‐transported fish. There are various ways this observation could be explained. Transported fish may have migrated slowly because they were recovering from the effects of transport and tagging. Alternatively, given that transported fish had advanced migration timing, they may have paused to more closely synchronize their movement phenology with that of in‐river migrants. Support is lent to the latter explanation by the lack of a marginal effect of transport on migration speed after accounting for any differences in migration timing between non‐transported and transported fish. This suggests that if the transported fish had been released when the in‐river migrants arrived, both groups of fish would have had similar migration speeds. Therefore, the slowed migration of transported fish was likely a function of their advanced migration timing rather than an inhibition of their capacity to migrate. Previous studies of *S. salar* smolts have documented no relationship between post‐transport recovery duration and migration rate (Thorstad et al., 2012b) and no effect of trapping and tagging on the rate, diel timing, and trajectory of migration (Sortland et al., 2024). These works provide further evidence that transport does not temporarily suppress migration.

The effect of transport on migration success downstream of the transported fish release site may depend on how much translocation changes migration timing. In agreement with the comparable migration success downstream of the transported fish release site observed here, hatchery‐reared *S. salar* can also have similar (30% vs. 33%) early marine migration success rates regardless of whether they are released at the marine‐freshwater interface or 9 km upriver (Thorstad et al., 2012b). However, the effect of trap and transport on post‐release migration success is date‐dependent for Columbia River Basin juvenile *O. tshawytscha*; transport has no effect late in the migration season but has a negative effect in the early and middle migration season (Eder et al., 2009). Further emphasizing the importance of phenology, Rechisky et al. (2012) found that releasing transported fish at the same time as in‐river fish were expected to arrive increased their freshwater and nearshore marine outmigration success by 75% compared with those released ca. 5 weeks earlier. As such, a negative effect of trap and transport on post‐release migration success may be contingent on transport substantially advancing migration progress and thereby desynchronising marine arrival timing from the onset of favorable conditions (Cushing, 1990; Muir et al., 2006; Otero et al., 2014). Therefore, shorter transport distances, such as those reported here and by Thorstad et al. (2012b), may not advance migration enough to cause a marine arrival timing mismatch. Muir et al. (2006) also argued that increased predation rates due to reduced growth may partially drive decreased transported fish post‐release migration success in the Columbia River Basin. However, here only smolts ≥130 mm fork length and ≥20 g wet mass were tracked, which may have masked any size‐specific mortality effect.

In this study, fish were tracked at the beginning of their first seaward migration, but transport may cause differential survival during later life stages, for example the marine survival effect size (Rechisky et al., 2012) and direction (Gosselin et al., 2021) of trap and transport can vary over space. Furthermore, transport can decrease adult salmonid upstream migration success (Gosselin et al., 2021) and increase within‐catchment straying (Mills, 1994; Tattam & Ruzycki, 2020), presumably by disrupting sequential exposure to olfactory cues during outmigration (Haraldstad et al., 2022; Keefer & Caudill, 2014). The limited existing capture‐mark‐recapture research indicates that trap and transport increases *S. salar* adult return rate (Mills, 1994) when fish are not highly stressed at release (Virtanen et al., 1991). Therefore, although trap and transport may not increase survival across all life stages, the positive effect of transport on early outmigration success could increase adult return rate relative to non‐transported fish.

## 5 CONCLUSION

In two study areas, *S. salar* smolts were trapped and transported around natural lakes, and the effects of this intervention on outmigration success, speed, and timing were estimated. Transport increased overall migration success from the capture site to the end of the river and from the capture site to the outer estuary. This effect was largely the product of high transport success; all fish survived transport, while in‐river migration success was relatively low between the capture site and the transported fish release site. Transport did not affect migration success downstream of the transported fish release site, indicating that translocating salmon smolts by road does not increase their post‐release migration failure rate above the level experienced by in‐river fish. However, in this study, fish were only tracked from freshwater to the outer limit of a marine inlet. Therefore, unobserved delayed transport effects may have occurred later in the migration. For example, the advanced migration timing of transported fish could have affected their offshore survival. The findings presented here indicate that trap and transport is an effective, though expensive and labor‐intensive, way to increase *S. salar* migration success during the early stages of their first seaward migration. However, long‐term use of the intervention on wild populations should be approached with caution. If transported fish are more likely to reproduce as adults, as appears to be the case based on differential return rates (Mills, 1994; Virtanen et al., 1991), then trap and transport will remove selection pressure for successful smolt migration. Over time, this could result in a population that is poorly adapted for the first seaward migration, and maladapted populations may be difficult to restore to a self‐sustaining, naturally migrating status (Lennox et al., 2021). Therefore, trap and transport should be used as either a last‐resort, long‐term solution that is only employed where restoration is impossible (such as in rivers heavily altered for active hydropower schemes), or as a short‐term response to some event, such as pollution or construction, that will temporarily substantially decrease migration success.

## AUTHOR CONTRIBUTIONS

The work was conceived and planned by B.A.S., P.R., M.F., and C.E.A. The fieldwork was conducted by R.F., H.M.H., J.L., A.G., J.R.R., B.S., P.R., J.P.K., C.W.B., and C.E.A. R.F. analyzed the data and wrote the manuscript with contributions and feedback from all authors.

## Supporting information


**Appendix S1.** Fish release dates.

**Appendix S2.** Receiver array detection efficiencies.

**Appendix S3.** Migration timing in the Lomond study area.
