# Supplementary material for: The effect of downstream translocation on Atlantic salmon Salmo salar smolt outmigration success
Source: J Fish Biol. 2024 Oct 12;106(2):376–88. doi: 10.1111/jfb.15928 (PMC11842170; doi:10.1111/jfb.15928)
Supplement: Supplementary file 1 — Appendix S1. Fish release dates. [file JFB-106-376-s002.docx]

**Appendix A – Fish release dates**

Table A1. Tagged Salmo salar smolt release dates.

| **Study area** | **Date (yyyy-mm-dd)** | **Non-transported fish released (n)** | **Transported fish released (n)** |
| --- | --- | --- | --- |
| Derwent | 2021-04-14 | 1 | 0 |
| Derwent | 2021-04-16 | 6 | 0 |
| Derwent | 2021-04-18 | 6 | 0 |
| Derwent | 2021-04-20 | 0 | 10 |
| Derwent | 2021-04-21 | 0 | 6 |
| Derwent | 2021-04-22 | 0 | 8 |
| Derwent | 2021-04-24 | 8 | 0 |
| Derwent | 2021-04-25 | 6 | 0 |
| Derwent | 2021-04-27 | 7 | 10 |
| Derwent | 2021-04-29 | 3 | 7 |
| Derwent | 2021-05-03 | 34 | 0 |
| Derwent | 2021-05-04 | 12 | 17 |
| Derwent | 2021-05-05 | 9 | 0 |
| Lomond | 2021-04-15 | 4 | 0 |
| Lomond | 2021-04-17 | 8 | 0 |
| Lomond | 2021-04-18 | 4 | 0 |
| Lomond | 2021-04-19 | 4 | 0 |
| Lomond | 2021-04-21 | 10 | 0 |
| Lomond | 2021-04-22 | 2 | 0 |
| Lomond | 2021-04-23 | 4 | 4 |
| Lomond | 2021-04-24 | 5 | 6 |
| Lomond | 2021-04-25 | 4 | 4 |
| Lomond | 2021-04-26 | 0 | 5 |
| Lomond | 2021-04-27 | 5 | 9 |
| Lomond | 2021-04-28 | 11 | 14 |
| Lomond | 2021-04-29 | 18 | 0 |
| Lomond | 2021-04-30 | 0 | 4 |
| Lomond | 2021-05-02 | 5 | 0 |
| Lomond | 2021-05-03 | 13 | 0 |
| Lomond | 2021-05-04 | 1 | 0 |
