# Supplementary material for: The effect of downstream translocation on Atlantic salmon Salmo salar smolt outmigration success
Source: J Fish Biol. 2024 Oct 12;106(2):376–88. doi: 10.1111/jfb.15928 (PMC11842170; doi:10.1111/jfb.15928)
Supplement: Supplementary file 2 — Appendix S2. Receiver array detection efficiencies. [file JFB-106-376-s003.docx]

**Appendix B – Receiver array detection efficiencies**

The detection efficiency of each receiver array in the study area was determined as the number of fish detected at and downstream of the array as a proportion of those detected downstream of the array (*sensu* Melnychuk, 2012). Only non-transported fish were used in the detection efficiency calculations for receivers located at and upstream of the transported fish release sites. Detection efficiency could not be estimated for receivers deployed in lakes or river sections with more than one channel nor could it be estimated for the final receiver array in each study area. The names and locations of receiver arrays with estimable detection efficiencies are given in Figure 1 and Figure 2. The detection efficiencies themselves are provided in Table 1 and Table 2.


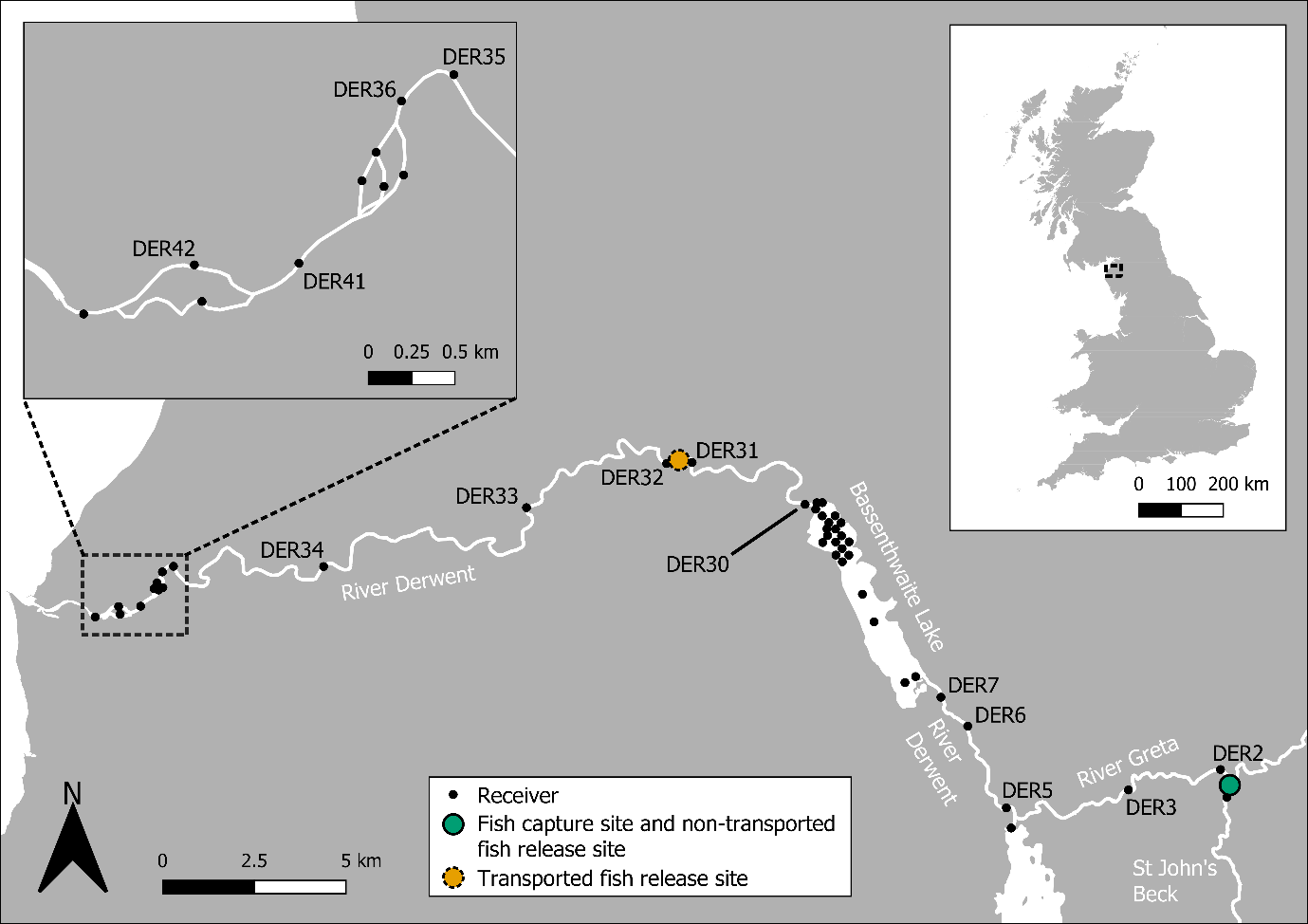


Figure B1. The Derwent study area. Receiver names are in black text and waterbody names are in white text. Only the names of receivers arrays with estimable detection efficiencies are given. This figure contains public sector information licensed under the Open Government Licence v3.0. Contains OS data © Crown copyright and database right 2021.

Table B1. Derwent study area receiver array detection efficiencies.

| Receiver array | Detection efficiency |
| --- | --- |
| DER2 | 0.508 |
| DER3 | 0.048 |
| DER5 | 0.532 |
| DER6 | 0.984 |
| DER7 | 0.984 |
| DER30 | 0.947 |
| DER31 | 0.763 |
| DER32 | 0.541 |
| DER33 | 1 |
| DER34 | 0.476 |
| DER35 | 0.889 |
| DER36 | 1 |
| DER41 | 1 |
| DER42 | 0.733 |


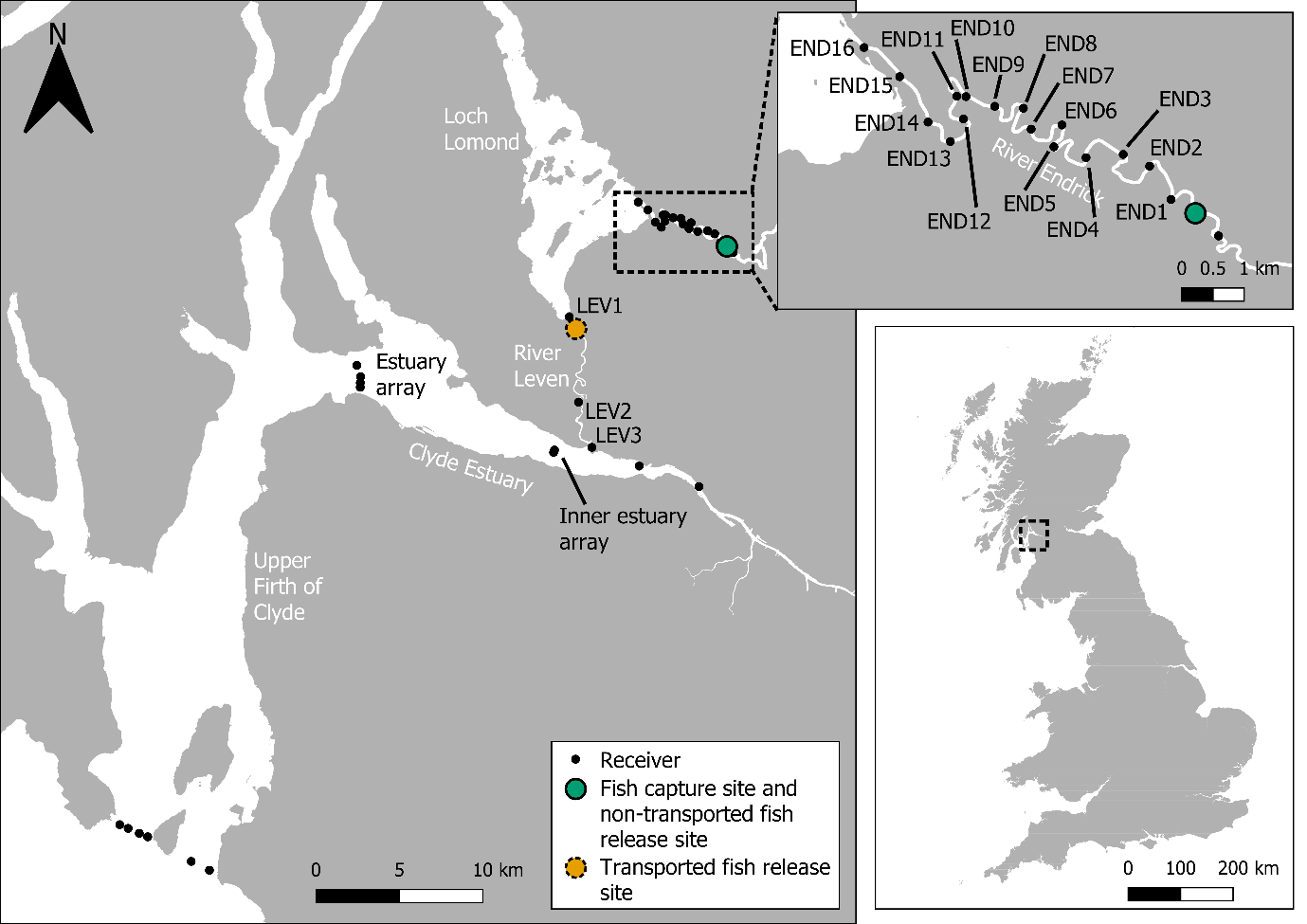


Figure B2. The Lomond study area. Selected receiver names are in black text and water body names are in white text. Only the names of receivers arrays with estimable detection efficiencies are given. Only receivers recovered after deployment are plotted. Contains public sector information licensed under the Open Government Licence v3.0. Contains OS data © Crown copyright and database right 2021.

Table B2. Lomond study area receiver array detection efficiencies.

| Receiver array | Detection efficiency |
| --- | --- |
| END1 | 0.978 |
| END2 | 0.941 |
| END3 | 0.706 |
| END4 | 0.847 |
| END5 | 0.952 |
| END6 | 0.976 |
| END7 | 0.988 |
| END8 | 0.976 |
| END9 | 0.988 |
| END10 | 1 |
| END11 | 1 |
| END12 | 0.988 |
| END13 | 1 |
| END14 | 1 |
| END15 | 1 |
| END16 | 1 |
| LEV1 | 0.591 |
| LEV2 | 0.76 |
| LEV3 | 1 |
| Inner Estuary Array | 0.778 |
| Estuary Array | 0.805 |

References

Melnychuk, M. C. (2012). Detection efficiency in telemetry studies: Definitions and evaluation methods. *Telemetry Techniques: A User Guide for Fisheries Research. Bethesda: American Fisheries Society*, 339–357.
