# Supplementary material for: The effect of downstream translocation on Atlantic salmon Salmo salar smolt outmigration success
Source: J Fish Biol. 2024 Oct 12;106(2):376–88. doi: 10.1111/jfb.15928 (PMC11842170; doi:10.1111/jfb.15928)
Supplement: Supplementary file 3 — Appendix S3. Migration timing in the Lomond study area. [file JFB-106-376-s001.docx]

**Appendix C – Migration timing in the Lomond study area**

The effect of transport on river exit timing depended on study area; there was a stronger advancement in the Derwent than the Lomond system (see section 3.2.1 of the manuscript). This interaction may have been explained by receiver layout differences between study areas. In the Derwent system the final river receiver was ~3.3 km upstream from the river mouth while in the Lomond system it was at the river mouth. If the fish paused before entering the estuary (Lilly *et al*., 2022), this may have happened after the final Derwent river receiver but before the final Lomond river receiver. If this pause was longer for transported than non-transported fish (as might be the case if the earlier arriving transported fish wait to enter the estuary with more co-migrants), then the migration timing difference would be larger upstream of the pause’s location.

To test this hypothesis, in the Lomond system, arrival timing at the river mouth (LEV3) and the next upstream (LEV2) was compared between transported and non-transported fish with a linear mixed effects model constructed in the R package lme4 (Bates *et al.*, 2015). It was assumed that LEV2, which was ~3.4km upstream of river mouth, was upstream of the hypothesised pause location. In the most complex model arrival Day of the Year (DOY) at LEV2 and LEV3 was the response variable while release DOY (continuous), receiver identity (LEV2 or LEV3) and migration route (non-transported or transported) were specified as fixed effects. Individual was included as a random intercept to account for the non-independence of repeated observations of individuals across receivers. The most complex model also included all possible pairwise interactions between the fixed effects. The stepwise backwards model selection procedure described in Section 2.4.1 of the manuscript was used to test the significance of all fixed effects in the most complex model.

Arrival DOY did not vary as a function of an interaction between receiver identity and migration route. This indicates that the effect of transport on arrival date was consistent across LEV2 and LEV3 (Figure C1) and does not support the hypothesis that differences in receiver layout among study areas were the driver of different-sized advancive transport effects. The only fixed effect in the final model was receiver identity (χ^2^ = 6.920, df = 1, p = 0.008). The model-predicted arrival date for LEV2 was DOY 115.187 while that for LEV3 was DOY 116.053. These predictions were for the median release DOY (115) and for the tag with the LEV2 arrival DOY nearest the median arrival DOY for that receiver.


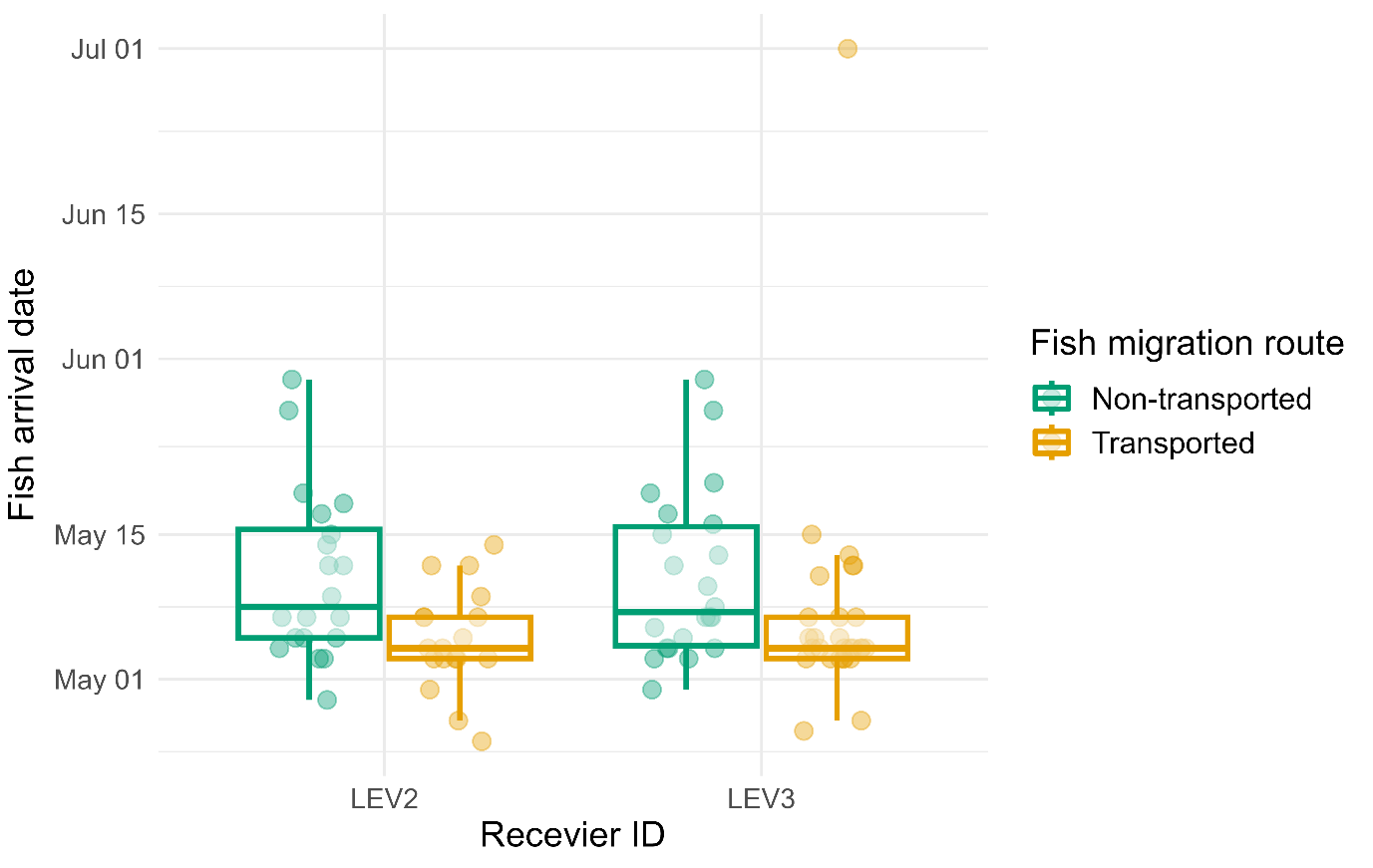


Figure C1. Salmo salar smolt arrival dates at the two most downstream freshwater receivers in the Lomond system. LEV2 was located ~3.3 km upstream of the river mouth while LEV3 was at the river mouth. In the boxplots, the lower hinge represents the first quartile, the middle horizontal line represents the median and the upper hinge represents the third quartile. The whiskers of the boxplots reach to the largest observed values that are at most 1.5 times the interquartile range from the upper or lower hinge. The points, which are horizontally jittered, mark the observed fish arrival dates.

References

Bates, D., Mächler, M., Bolker, B., & Walker, S. (2015). Fitting linear mixed-effects models using **lme4**. *Journal of Statistical Software*, *67*(1). <https://doi.org/10.18637/jss.v067.i01>

Lilly, J., Honkanen, H. M., Bailey, D. M., Bean, C. W., Forrester, R., Rodger, J., & Adams, C. E. (2022). Investigating the behaviour of Atlantic salmon (*Salmo salar* L.) post‐smolts during their early marine migration through the Clyde Marine Region. *Journal of Fish Biology*, *101*(5), 1285–1300. <https://doi.org/10.1111/jfb.15200>
